# CD81 Enhances Radioresistance of Glioblastoma by Promoting Nuclear Translocation of Rad51

**DOI:** 10.3390/cancers13091998

**Published:** 2021-04-21

**Authors:** Wang Zheng, Qianping Chen, Hongxia Liu, Songling Hu, Yuchuan Zhou, Yang Bai, Jianghong Zhang, Yan Pan, Chunlin Shao

**Affiliations:** Institute of Radiation Medicine, Shanghai Medical College, Fudan University, Shanghai 200032, China; 17211140008@fudan.edu.cn (W.Z.); 17111140001@fudan.edu.cn (Q.C.); 18111140003@fudan.edu.cn (H.L.); 18111140002@fudan.edu.cn (S.H.); 18211140009@fudan.edu.cn (Y.Z.); yangbai@fudan.edu.cn (Y.B.); zjh551268@fudan.edu.cn (J.Z.); swallowpan@fudan.edu.cn (Y.P.)

**Keywords:** glioblastoma, radioresistance, CD81 regulation, Rad51 nuclear translocation

## Abstract

**Simple Summary:**

CD81 is highly expressed in glioblastoma (GBM) as a transmembrane protein. The functional study demonstrated that CD81 contributed to radioresistance of GBM. Further evidence showed that CD81 expression was closely related to DNA damage response and homologous recombination repair (HRR) was responsible for the CD81 mediated radioresistance. Particularly, nuclear membrane protein CD81 assisted the nuclear transport of Rad51, a key protein involved in HRR process after irradiation. Overall, CD81 may be utility as a predictive biomarker and therapeutic target of radioresistant GBM.

**Abstract:**

Glioblastoma (GBM) is the most common type of primary tumor in central nervous system in adult with a 5-year survival rate of ≤5%. Despite of recent advances in tumor radiotherapy, the prognosis of GBM remains to be dismal due to radioresistance. In this study, we identified CD81 as a potential biomarker of GBM radioresistance with the analysis of upregulated genes in human glioma radioresistant cell lines U251R and T98G in comparison with U251 cells. In vitro and in vivo experiments demonstrated that suppressing CD81 by siRNA/shRNA enhanced radiation-induced cell killing and DNA damage of γ-H2AX formation, and delayed tumor xenograft growth of GBM. Mechanistically, we found that knockdown of CD81 significantly decreased radiation-induced expression of nuclear Rad51, a key protein involved in homologous recombination repair (HRR) of DNA, suggesting that CD81 is essential for DNA damage response. Meanwhile, when the cells were treated with B02, a Rad51 inhibitor, silencing CD81 would not sensitize GBM cells to radiation, which further illustrates that Rad51 acts as an effector protein of CD81 in tumor radioresistance. Dual immunofluorescence staining of CD81 and Rad51 illustrated that nuclear membrane CD81 contributed to the nuclear transport of Rad51 after irradiation. In conclusion, we demonstrated for the first time that CD81 not only played a vital role in DNA repair through regulating Rad51 nuclear transport, but also might serve as a potential target of GBM radiotherapy.

## 1. Introduction

Glioblastoma (GBM) is the most common lethal brain cancer and has been classified into the grade IV glioma with extremely high malignancy by WHO [1]. The current therapeutic modality for GBM is neurosurgical resection followed by radiotherapy and/or chemotherapy [2]. Despite of the multimodality therapy, the life expectancy for GBM patients remains extremely poor with a median survival of 14.6 months [3,4]. Growing evidence has shown that radioresistance of GBM patients is partially responsible for the limited therapy effect [5]. Although several mechanisms have been proposed to explain the radioresistance of GBM [6,7,8], the molecular basis of radioresistance remains incompletely defined.

In order to identify novel biomarker of GBM radioresistance, we performed Tandem Mass Tag (TMT) analysis on three GBM cell lines and screened out a list of differential expression genes (DEGs) with a variety of functions in radioresistance. Analysis of these DEGs using public clinical databases revealed for the first time that CD81 could be a novel regulator of radioresistance. CD81 is a member of the transmembrane 4 superfamily (TM4SF) and can regulate the entry of hepatitis C virus (HCV) into liver cells [9]. As a molecule located on various membrane structures, it is of great concern how CD81 promotes resistance to ionizing radiation.

Among all mechanisms involved in radioresistance, autophagy and DNA damage responses (DDRs) are considered to be the most active regulations irrespective of pathological grades of cancer, and they could be induced in nearly all GBMs after radiotherapy and/or temozolomide (TMZ) treatment [10,11]. Regulation of autophagy contributes to either radiosensitization or radioresistization under different conditions. Some studies showed that inhibition of autophagy by chloroquine increased the sensitivity of GBM to ionizing radiation (IR) [12,13]. Whereas other investigators found that GDC-0941, a known autophagy inducing agent, drastically increased the sensitization of GBM to the combination treatment of IR and temozolomide [14]. The double-edged sword role of autophagy in cancer radiotherapy are still debatable. While since serious DNA damage is the main cause of radiation-induced cell death, it is unequivocal that the enhancement of DDRs results in tumor resistance to radiation [15,16,17]. Thus, it is of great importance to further disclose the molecular mechanisms of DDRs and GBM radioresistance. Our current findings have revealed novel roles of CD81 in DDRs and radioresistance of GBM, suggesting that CD81 may represent a sensitization target for GBM radiotherapy.

## 2. Results

### 2.1. CD81 Had Close Relevance with GBM in Clinic

**Figure 1:**
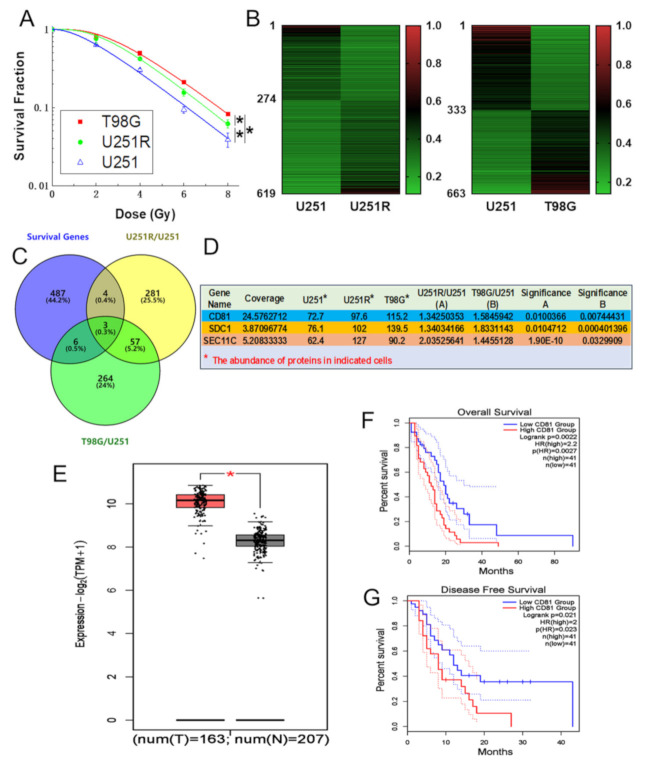
CD81 had close relevance with GBM in clinic. (**A**) Clonogenic survivals of T98G, U251R, and U251 cells exposed to 0, 2, 4, 6, and 8 Gy of X-rays. (**B**) Heat maps of the differential expression genes between U251 and U251R cells (left), T98G and U251 cells (right) (fold change ≥ 1.2, *p* < 0.05). (**C**) Wayne chart of the overexpressed genes in U251R and T98G cells in comparison with U251 cells, which significantly impacted the prognosis of GBM patients (*p* < 0.05). The intersection contained three genes. Yellow and green parts represented the upregulated genes in U251R and T98G cells, respectively. Blue part represented the survival-related genes of GBM patients (data from GEPIA). (**D**) The expression levels of above three genes in U251, U251R, and T98G cells. (**E**) Average level of CD81 in 163 GBM tissues (T) and 207 normal tissues (N) based a GEPIA database. (**F**) Kaplan–Meier analysis of the relationship between CD81 expression and overall survival (OS) of GBM patients (data from GEPIA). (**G**) Kaplan–Meier analysis of the relationship between CD81 expression and disease-free survival (DFS) of GBM patients (data from GEPIA). *, *p* < 0.05 between indicated groups.

Our previous work has successfully established a radioresistant cell line (named as U251R) from human glioma U251 cells and issued the DEGs between U251 and U251R cells by TMT quantitative proteomic analysis [18]. To know much more genes precisely involved in the radioresistance of GBM, here we further explored the DEGs between U251 and T98G cells by TMT analysis. T98G is a kind of glioblastoma multiforme cells and had a radioresistance much higher than U251R and U251 cells (Figure 1A). In comparison with U251 cells, U251R cells have an acquired radioresistance and T98G cells have an innate radioresistance. According to the TMT assay, there were 274 downregulated genes and 345 upregulated genes in U251R cells while 333 downregulated genes and 330 upregulated genes in T98G cells (Figure 1B). On the other hand, we gathered 500 most high expressed survival-relative genes in GBM patients derived from GEPIA database (website: http://gepia2.cancer-pku.cn on 20 May 2020) and then compared them with the above 345 upregulated genes in U251R and 330 upregulated genes in T98G cells. Three commonly high-expressed genes were identified in the cross-section (Figure 1C), where CD81 had the highest coverage (the percentage of the protein sequence covered by identified peptides) (Figure 1D). The database mineral resource analysis demonstrated that CD81 had close clinical relevance, i.e., the overexpression of CD81 was significantly related to the GBM tumorigenesis (Figure 1E). Moreover, according to the Kaplan–Meier analysis of overall survival (OS) and disease-free survival (DFS), the prognosis of GBM patients with high expression level of CD81 was markedly worse than that with low level of CD81 (Figure 1F,G). These bioinformatic analyses indicate that CD81 may serve as a candidate biomarker of tumor radioresistance and therapeutic outcome.

### 2.2. Inhibition of CD81 Enhanced Radiosensitivity of GBM Cells In Vitro and In Vivo

**Figure 2:**
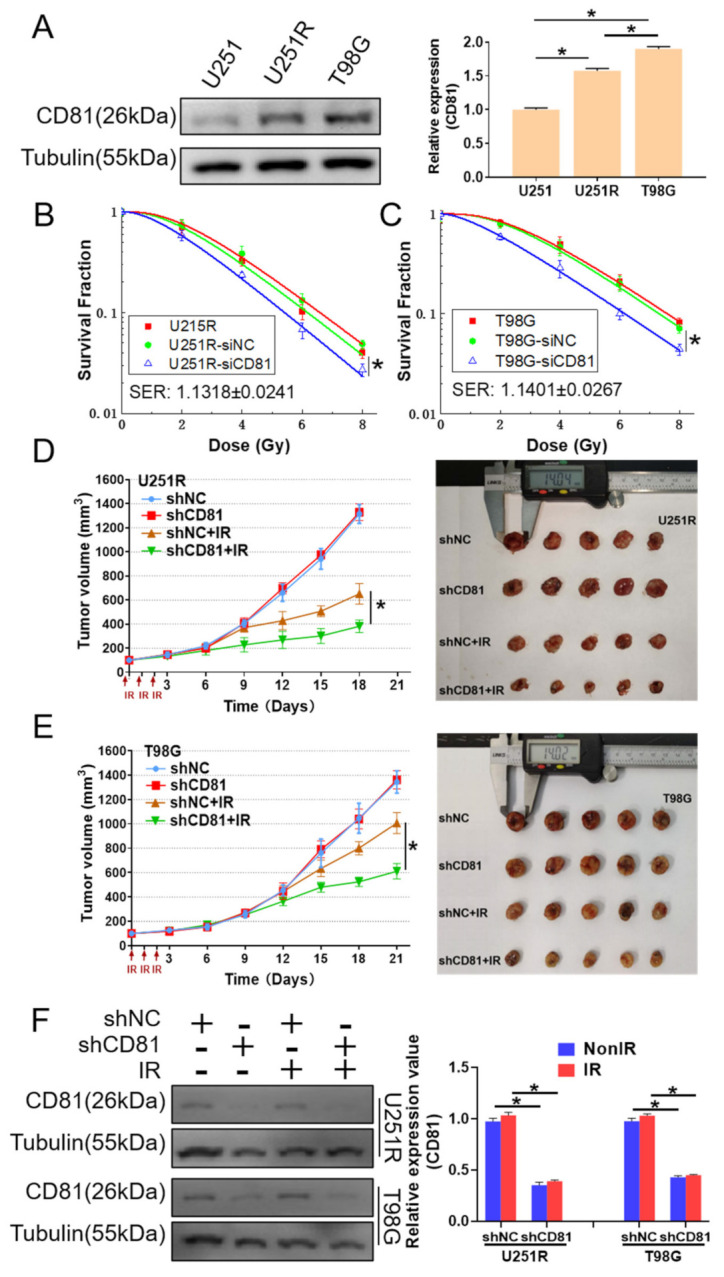
Inhibition of CD81 enhanced radiosensitivity of GBM cells in vitro and in vivo. (**A**) Western blot analysis (**left**) and quantitation (**right**) of CD81 expression level in U251, U251R, and T98G cells. Tubulin was used as a loading control. (**B**,**C**) Clonogenic survivals of U251R cells (**B**) and T98G (**C**) cells transfected with siCD81 or siNC after X-ray irradiation. Sensitizer enhancement ratio (SER) was calculated with OriginLab software. (**D**,**E**) Growth curves of the xenograft tumors of U251R (**D**) and T98G (**E**) cells transfected with shCD81 or shNC with or without 3 × 8 Gy fractionated X-ray irradiation. Tumor volume was measured every three days with a digital caliper and calculated using the formula (L × W^2^) × π/6. Images of the dissected tumors from athymic nude mice (*n* = 5) were also shown. (**F**) Western blot analysis (left) and quantitation (right) of CD81 expression level in dissected tumors above. *, *p* < 0.05 between indicated groups.

To further disclose the relationship between CD81 and radiosensitivity of GBM, we detected the cellular expression of CD81 by Western blot assay and found that the expression of CD81 increased orderly in U251, U251R, and T98G cells (Figure 2A), which has a positive correlation with the radiosensitivity of these cells as shown in Figure 1A. When U251R and T98G cells were transfected by CD81 siRNA (siCD81) with an effective interfering efficiency verified by Western blot assay (Supplementary Figure S1), their survival fractions were obviously lower than that of cells transfected with negative control siRNA (siNC) (Figure 2B,C). Therefore, transfection of siCD81 markedly sensitized GBM cells to irradiation.

Moreover, we transfected U251R and T98G cells with CD81 shRNA (shCD81) to establish the stable CD81-knockdown GBM cells and further investigated the influence of CD81 on tumor radioresistance in vivo. The effective interfering efficiency of shCD81 was also shown in Supplement Figure S1. U251R and T98G cells transfected with shNC and shCD81 were subcutaneously injected into athymic nude mice. When the tumor approached to about 100 mm^3^, they were locally irradiated with fractional doses of 8 Gy/day in three consecutive days and then the growth curves of xenografts were observed. Consistent with the in vitro experimental results, knockdown of CD81 further enhanced radiation-induced growth suppression of glioma xenografts of both U251R and T98G cells (Figure 2D,E). Tumor growth delayed (TGD) of shNC + IR and shCD81 + IR treatments was 4.615 and 9.597 d for U251R cells, 0.424 and 3.882 d for T98G cells, respectively. Moreover, we detected CD81 expression in dissected tumors and confirmed that CD81 was significantly down-regulated in shCD81 transfected GBM cells in vivo (Figure 2F). Accordingly, the intracellular high level of CD81 contributes to the radioresistance of GBM.

### 2.3. Downregulation of CD81 Increased DNA Damage and Decreased DNA Repair

**Figure 3:**
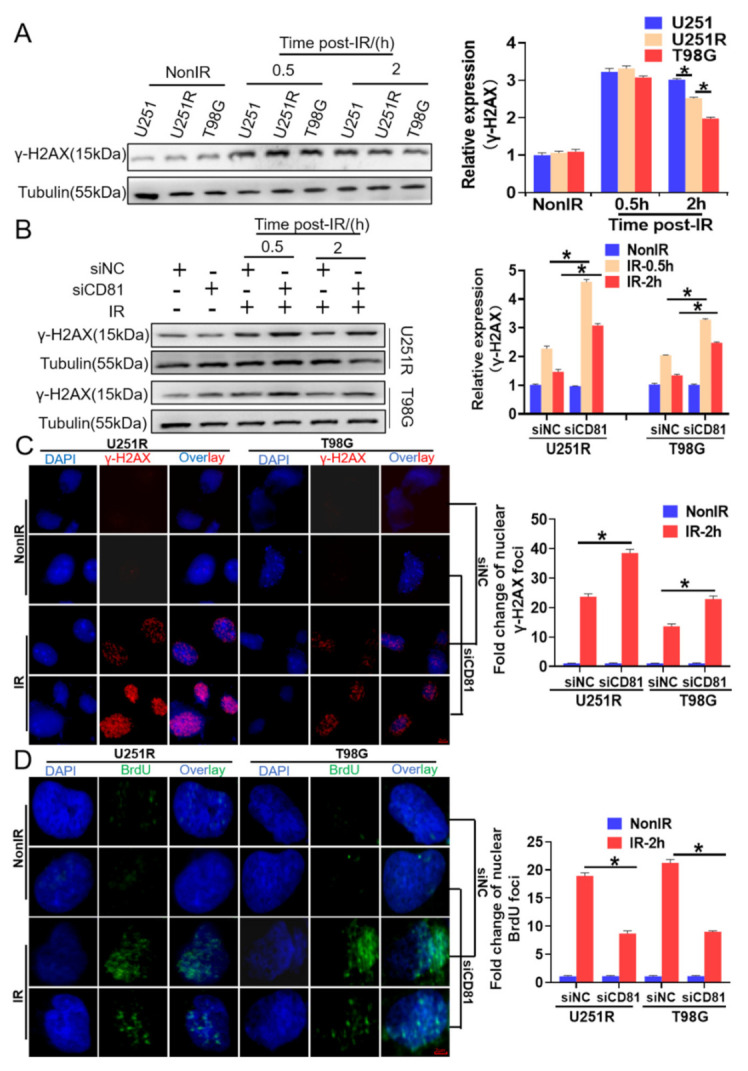
Downregulation of CD81 increased DNA damage and decreased DNA repair in the irradiated cells. (**A**) Western blot assay (**left**) and quantitation (**right**) of γ-H2AX level in U251, U251R, and T98G cells at indicated time after 6 Gy irradiation. (**B**) Influence of siCD81 on the formation of γ-H2AX in 6 Gy irradiated U251R and T98G cells transfected with siCD81 and siNC, respectively. (**C**) Immunostaining images (**left**) and quantification (**right**) of γ-H2AX foci in U251R and T98G cells at 2 h after irradiation. Blue, DAPI stained nuclear. Red, γ-H2AX foci. (**D**) Immunostaining images (**left**) and quantification (**right**) of BrdU foci in U251R and T98G cells at 2 h after irradiation. Blue, DAPI stained nuclear. Green, BrdU foci. *, *p* < 0.05 between indicated groups.

As DNA damage repair is recognized as a vital mechanism in tumor radioresistance and γ-H2AX was regarded as a marker of DNA damage, we detected the levels of γ-H2AX in U251, U251R, and T98G cells exposed to 6 Gy X-rays irradiation. It was found that the γ-H2AX protein levels were obviously increased by irradiation but there was no significant difference among these cell lines at 0.5 h after IR, indicating that radiation-induced initiation DNA damage were similar in these cells. At 2 h after IR, the γ-H2AX protein levels were still at high levels but decreased orderly in U251, U251R, and T98G cells (Figure 3A), indicating the DNA damage repair ability increased with radiation resistance of GBM cells.

To know the relationship of DNA damage and CD81 expression, we detected the γ-H2AX levels in U251R and T98G cells transfected with siCD81 after 0.5 or 2 h of IR. Figure 3B illustrates that inhibition of CD81 significantly enhanced the expression of radiation-induced γ-H2AX. Moreover, we visualized γ-H2AX foci by immunofluorescence assay and found that the nuclear γ-H2AX foci accumulation was also significantly increased in siCD81-transfected GBM cells in comparison with siNC group at 2 h after 6 Gy IR (Figure 3C).

Afterwards, we wonder whether the suppress effect of CD81 on DNA damage is relative to DNA repair, especially homologous recombination repair (HRR), an error-free repair. To test this, we take advantage of the fact that the incorporation of BrdU into genome is detectable by anti-BrdU antibody only in the region of ssDNA, a substrate for HRR [19]. The cells were cultured in the medium containing 20 μM BrdU for 36 h and then irradiated with 6 Gy X-rays. After 2 h of irradiation, the cells were fixed and stained with anti-BrdU antibody. It was found that the foci number of BrdU was extensively increased in the siNC-transfected cells, which was about twofold of that in the siCD81-transfected cells (Figure 3D), suggesting that CD81 silence leads to a defection of HRR-dependent ssDNA generation.

In addition, we performed flow cytometry assay to assess the effect of CD81 inhibition on G2/M-phase arrest, a cell cycle regulation which has great benefit to HRR in GBM cells. We found that, after 6 Gy irradiation, the percentage of G2/M-arrested population in the siCD81-transfected cells was lower than that in the siNC-transfected cells (8.1% vs. 21.3% in U251R cells; 9.68% vs. 17.68% in T98G cells) (Supplement Figure S2A,B), which further strengthens the deduction that CD81 contributes to HRR in overcoming IR-induced DNA damage in GBM cells.

### 2.4. Suppress of CD81 Reduced Nuclear Translocation of Rad51

**Figure 4:**
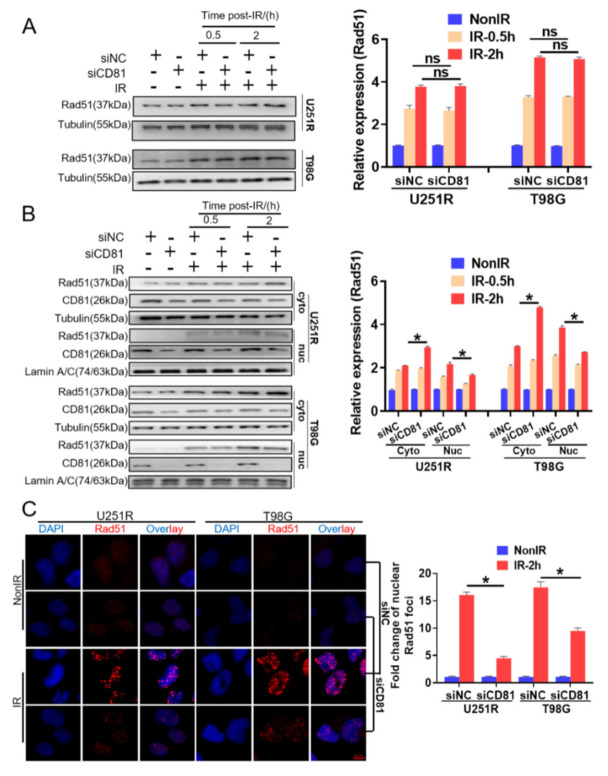
Suppression of CD81decreased nuclear translocation of Rad51 after 6 Gy irradiation. U251R and T98G cells were transfected with siNC and siCD81, respectively. (**A**) Western blot analysis (**left**) and quantitation (**right**) of cellular Rad51 level at 0.5 and 2 h post-IR. (**B**) Western blot analysis (**left**) and quantitation (**right**) of cytoplasmic (cyto) and nuclear (nuc) Rad51 level at 0.5 and 2 h post-IR. (**C**) Immunostaining images (left) and quantification (right) of nuclear Rad51 foci at 2 h post-IR. Blue, DAPI stained nuclear. Red, Rad51 foci. *, *p* < 0.05 between indicated groups. ns, no significant difference (*p* > 0.05 between indicated groups).

Given the critical role of CD81 in DNA damage repair, we further examined whether CD81 inhibition affected the expression of Rad51, a key protein of HRR. However, Western blot assay showed that suppression of CD81 in GBM cells could not reduce the cellular level of Rad51 neither at 30 min nor 2 h post-IR (Figure 4A). Considering that the translocation of Rad51 from cytoplasm to nuclear is necessary for its function in HRR, we wonder whether CD81 inhibition affects Rad51 by blocking its nuclear transport instead of reducing its cellular expression. To determine this, nuclear and cytoplasmic protein lysates were prepared from U251R and T98G cells that had been transfected with siNC or siCD81, respectively. It was found that the level of nuclear Rad51 increased in a time-dependent manner after irradiation, but CD81 inhibition markedly impaired this Rad51 accumulation in nuclear and correspondingly increased its cytoplasmic deposition (Figure 4B). Moreover, immunofluorescence assay also illustrated that, at 2 h post-IR, the nuclear accumulation of Rad51 foci in siCD81-transfected GBM cells were obviously lower than that in siNC-transfected cells (Figure 4C). This decrease of Rad51 foci due to CD81 inhibition was associated with both the delayed resolution of IR-induced γ-H2AX foci (Figure 3C) and the reduction of HRR in the siCD81-transfected cells (Figure 3D).

### 2.5. Rad51 Was Indispensable for CD81-Regulated Radioresistance

**Figure 5:**
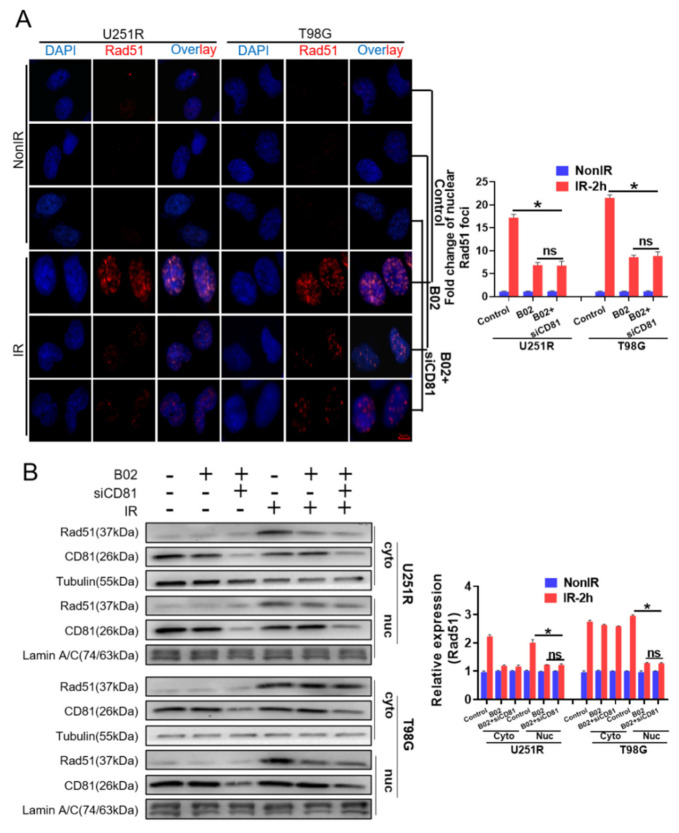
Effect of B02 treatment on Rad51 expression. U251R and T98G Cells were treated with B02 (10 μM), its control (1‰ DMSO), or B02 + siCD81 and then irradiated with X-rays. (**A**) Representative immunostaining images (**left**) and quantification (**right**) of nuclear Rad51 foci in the cells at 2 h after 6 Gy irradiation. Blue, DAPI stained nuclear. Red, Rad51 foci. (**B**) Western blot assay (**left**) and quantitation (**right**) of cytoplasmic (cyto) and nuclear (nuc) proteins of Rad51 level and CD81 at 2 h after 6 Gy irradiation. *, *p* < 0.05 between indicated groups. ns, no significant difference (*p* > 0.05 between indicated groups.

**Figure 6:**
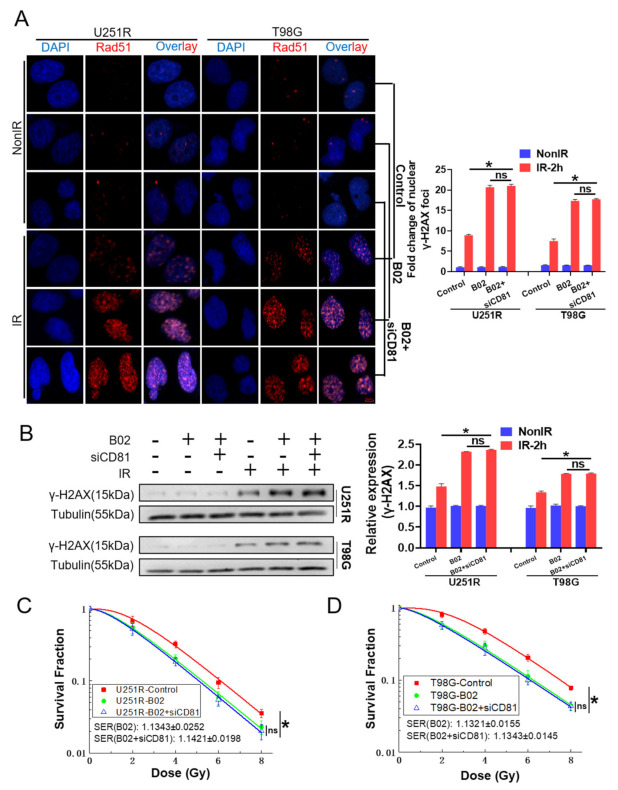
Rad51 was indispensable for CD81-regulated radioresistance. U251R and T98G Cells were treated with B02 (10 μM), its control (1‰ DMSO), or B02 + siCD81 and then irradiated with X-rays. (**A**) Representative immunostaining images (**left**) and quantification (**right**) of γ-H2AX foci in the cells at 2 h after 6 Gy irradiation. Blue, DAPI stained nuclear. Red, γ-H2AX foci. (**B**) Western blot assay (**left**) and quantitation (**right**) of γ-H2AX level at 2 h after 6 Gy irradiation. (**C**,**D**) Clonogenic survivals of X-ray irradiated U251R (**C**) and T98G (**D**) cells under different treatment of B02 or B02 + siCD81. *, *p* < 0.05 between indicated groups. ns, no significant difference (*p* > 0.05 between indicated groups).

Next, we explored whether the inhibition of Rad51 nuclear translocation fully contributed to the CD81 inhibition-reduced radioresistance. Thus, we treated U251R and T98G cells with B02, a specific inhibitor of Rad51 recombinase, or B02 combined with siCD81 transfection, and then exposed the cells with 6 Gy X-rays. Although B02 and siCD81 transfection reduced nuclear Rad51 level and increased γ-H2AX in GBM cells, the levels of nuclear Rad51 and γ-H2AX foci between B02 group and B02 + siCD81 group had no significant difference at 2 h post-IR (Figure 5A and Figure 6A). Western blot analysis of their protein expressions showed the same results (Figure 5B and Figure 6B). Moreover, clonogenic survival assay demonstrated that the sensitizer enhancement ratio (SER) of B02 treatment on cell survival was almost the same with the SER of B02 plus siCD81 (1.1343 ± 0.0252 vs. 1.1421 ± 0.0198 for U251R cells; 1.1321 ± 0.0155 vs. 1.1343 ± 0.0145 for T98G cells) (Figure 6C,D), suggesting that Rad51 was an indispensable effector of CD81-mediated radioresistance in GBM cells.

### 2.6. Nuclear Membrane CD81 Promoted Nuclear Transport of Rad51

**Figure 7:**
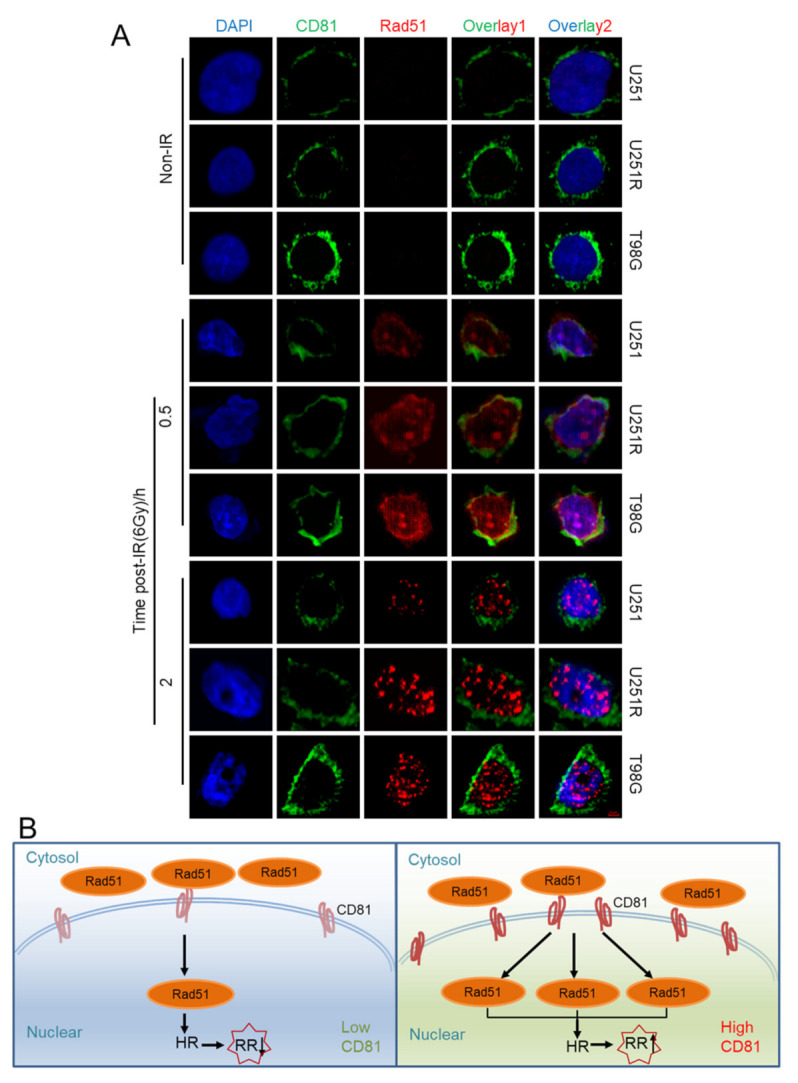
Nuclear membrane protein CD81 promoted nuclear transport of Rad51. (**A**) Immunofluorescence images of CD81 and Rad51 distribution in U251, U251R, and T98G cells at 0.5 and 2 h after 6 Gy irradiation. Blue, DAPI stained nuclear. Green, CD81 expression. Red, Rad51 foci. Overlay 1 represents the merged images of CD81 and Rad51 fluorescence, overlay 2 represents the merged images of overlay 1 and DAPI. (**B**) The diagrammatic sketches of the relationship between the level of nuclear membrane protein CD81 and nuclear translocation of Rad51 in GBM cells. High level of CD81 promotes the entry of Rad51 to nuclear, thus enhances homologous recombination repair ability and consequently raises tumor radioresistance.

Given that CD81 exists in almost all membrane structure and functions as a transmembrane movement mediator, we then investigated the potential mediator role of CD81 in Rad51 nuclear transport. At different time points after irradiation, CD81 and Rad51 proteins were stained in U251, U251R, and T98G cells. Figure 7A illustrates that, at 30 min post-IR, Rad51 was mainly detected around the nuclear where CD81 located, and few Rad51 foci were formed inside of nuclear. However, at 2 h post-IR, a large amount of Rad51 foci were translocated from nuclear membrane to intra-nucleus. Furthermore, nuclear Rad51 foci in U251, U251R, and T98G cells were increased orderly, consistent with the CD81 levels in these cell lines. Therefore, Rad51 could translocate into nuclear in a time-dependent manner with the assistance of CD81 during HRR of GBM cells (Figure 7B).

## 3. Discussion

Our data revealed the interaction of CD81-Rad51 in regulating DNA repair of irradiated GBM cells. Specifically, the CD81 expression was upregulated in the radioresistant GBM cells. Besides, the clinical database analysis illuminated a positive correlation between high level of CD81 and tumorigenesis as well as poor prognosis in GBM patients. Transcriptional inhibition of CD81 using siRNA and shRNA significantly enhanced radiosensitivity of GBM in vitro and in vivo, respectively. Mechanistically, CD81 knockdown markedly increased DNA damage accumulation and decreased DNA repair through attenuating Rad51 nuclear translocation.

CD81, a member of the tetraspanin family, has been implicated in multiple diseases. Emphasis was first given to CD81 on its receptor role in the HCV invasion on liver cells. Pileri et al. reported that CD81 was imperative in binding the major envelope protein (E2) of HCV and mediating HCV attachment to the target cells [20]. Besides promoting HCV transfection, CD81 has a predicting value in cancer development. It was reported that, through the study of 225 newly-diagnosed multiple myeloma patients, patients harboring more CD81+ in tumor cell plasma had a less differentiation state, dismal survival, and higher chemoresistance [21]. In breast cancer, suppression of CD81 using shRNA resulted in the decrease of cell migration in vitro and led to the reduction of primary tumor growth, extravasation and lung metastasis in vivo [22]. Meanwhile, recent studies have disclosed the role of CD81 in cancer immunomodulation. It was demonstrated that the diminishment of CD81 in regulatory T cells and innate myeloid-derived suppressor cells interfered their recruitment to tumor microenvironment and thus limited immune escape and metastatic dissemination of tumor cells in CD81-deficient mice [23]. A series of experimental or clinical results have demonstrated that CD81 is a potential target for tumor therapy. However, studies on the role of CD81 in tumor radioresistance are hitherto lacking. Our study not only revealed that CD81 upregulation promoting GBM malignancy and radiotherapy resistance, but also suggested that inhibition of CD81 may be a potential radiotherapeutic strategy for GBM patients.

Moreover, our present study proposed a new concept that nuclear membrane CD81 is a mediator of nuclear translocation of Rad51. Rad51 is known to be involved in homologous recombination and DNA repair by mediating the homologous strand exchange [24]. In general, with IR stress, Rad51 could be transported from cytoplasm to nuclear [25] with assistance of many factors such as BRCA1 [26], BRCA2 [27], P53 [28], and STAT5a/b [29]. However, no correlation between Rad51 and transmembrane protein CD81 has been reported yet. In this study, we established the linkage between CD81 and Rad51 for the first time. This speculation derived from the findings that decreased Rad51-dependent DNA repair and G2/M arrest occurred in CD81 silenced GBM cells after exposed to 6 Gy of X-rays. Mechanistic association of Rad51 and CD81 in regulating HRR and radioresistance was demonstrated with immunofluorescence staining and cell survival assay, where the reduced amount of Rad51 in nuclear and radioresistance of GBM cells by CD81 inhibition were abolished by pharmacologic suppression of Rad51. In addition, Rad51 and CD81 were co-located at nuclear membrane and then separated within two hours during HRR process (Figure 7A), suggesting a transmembrane mediator role of CD81 in the nuclear translocation process of Rad51. Accordingly, the constitutive high expression level of CD81 could promote nuclear translocation of Rad51, enhance HRR, and ultimately result in radioresistance of GBM cells (Figure 7B).

Radiotherapy eliminates tumor cells due to its ability of inducing severe DNA damage [30]. Unfortunately, many kinds of tumors are resistant to radiotherapy with the facility of powerful DDR system [31,32]. According to the source of resistance, tumor radioresistance can be classified into the acquired resistance and the innate resistance [33]. This study found that HRR played an important role in both styles of radioresistance. Concretely, we established U251R cells by treating U251 cells with fractionated radiation doses with an accumulation dose of 60 Gy, which is considered as the acquired radioresistant GBM cell line. While T98G, a grade IV glioma cell characterized with high radioresistance, is regarded as the innate radioresistant GBM cell line. Our study demonstrated that suppressing HRR with a Rad51 inhibitor B02 markedly reduced the radioresistance of both U251R and T98G cells. Extending this result to clinical application, we believe that inhibition of HRR may be an ideal strategy for GBM treatment either in newly-diagnosed patients or radiation-relapsing patients, which is also supported by other reports. Vendrely et al. found that, by unbalancing DNA repair response, resveratrol and capsaicin could enhance the killing effect of IR on pancreatic ductal adenocarcinoma [34]. Efimova et al. found that pitavastatin, an HMG-CoA reductase inhibitor, could delay DNA repair and promoted senescence after IR exposure in breast cancer and melanoma [35].

On the other hand, identification of new strategies of sensitizing GBM to radiotherapy would not only enhance tumor killing responses, but also reduce radiation injury to the normal tissues surrounding glioma by cutting down the dose of radiotherapy properly. According to our findings, since CD81 promotes Rad51-dependent DNA repair and thus acts as an intrinsic factor of tumor radioresistance, we speculate that combination of radiotherapy and CD81 inhibitor may promote therapeutic effect by attenuating HRR in GBM. However, no clinical application of CD81 inhibition in cancer has been explored. Unlike the antigens such as CD20, CD19, or CD37 used to target tumor cells since they are expressed on specific tumor tissues and subpopulations, CD81 is widely expressed in both normal tissues and tumor cells [36,37,38]. This brings a challenge for CD81 antibody-based treatment due to potential off-target effects and non-specific toxicities, although one study has demonstrated no evident toxicity in cynomolgus monkeys administered with a humanized anti-CD81 monoclonal antibody (mAb) [39]. Moreover, recent research found that one particular anti-CD81 antibody, 5A6 (mouse IgG1), could induce direct killing effect on human B lymphoma cells in vitro and activated innate immune cytotoxic mechanisms in vivo [40]. Therefore, we are eager to find an anti-CD81 mAb or relative drug which could specifically target CD81 on GBM cells.

## 4. Materials and Methods

### 4.1. Cell Culture and Irradiation

Human glioblastoma cell lines of U251 and T98G were purchased from Cell Bank of Chinese Academy of Science. The stable U251R cell line was previously developed from its parental cell line U251 by exposing it with 2 Gy X-ray/day for 30 fractions (5 fractions/weekly in general) with a total dose of 60 Gy [18]. U251 and U251R cells were cultured in DMEM medium (Gibco, Thermo Fisher Scientific, Waltham, MA, USA), and T98G cells were cultured in MEM medium (Gibco). All media were supplemented with 10% of fetal bovine serum and 100 units/mL of penicillin and 100 mg/mL of streptomycin (Gibco). Cells were incubated at 37 °C in 5% CO_2_ and subcultured every 3 days.

Cells in log-phase were irradiated with a dose rate of 0.883 Gy/min X-ray (X-RAD 320, PXI inc., North Branford, CT, USA; 12 mA, 2 mm aluminum filtration) at room temperature.

### 4.2. Tandem Mass Tag (TMT) Quantitative Proteomic Analysis

Total proteins were extracted from U251, U251R and T98G cells and their concentrations were detected with the bicinchoninic acid assay (BCA Protein Assay Kit; Beyotime Biotechnology, Haimen, China). A quantity of 0.2 mg of protein from each sample was used for TMT analysis following the manufacturer’s protocol (Genechem, Shanghai, China). A high-resolution mass spectrometer Q Exactive plus (Thermo Fisher Scientific) was used to perform the quantitative proteomics analysis. Protein sequence analysis was performed according to database Uniprot_HomoSapiens_20386_20180905 (website: http://www.uniprot.org accessed on 19 March 2019).

### 4.3. Clonogenic Assay

Radiosensitivity discrepancies among different cell lines were validated by the clonogenic survival assay. Briefly, cells were trypsinized and pipetted to single-cell suspension. Proper number of cells were planted in 6-well plates and allowed to grow overnight, then exposed to 0, 2, 4, 6, and 8 Gy of X-rays. These cells were cultured for another two weeks and then fixed for colony accounting. The number of colonies with more than 50 cells was counted. Survival (S) data after a radiation dose (D) are fit by a non-linear regression according to the single-hit multi-target formula S(D)/S(0) = 1 − (1 − exp(−D_0_/D))^n^ using origin software (OriginLab, version 9.5.1), where n has been interpreted as the number of distinct and identical targets that must be all inactivated to kill the cell, and D_0_ is the dose at which about 37% survival occurs. Sensitizer enhancement ratio (SER) is calculated as D_0_(control)/D_0_(treatment). To test statistically the difference between two different curves, the fitting data comparison function in the origin software was used.

### 4.4. Transient Transfection of siRNA

CD81 siRNA (target sense: 5′-AATTGAAGACGAAGAGCAG-3′) and its negative control (target sense: 5′-TTCTCCGAACGTGTCACGT-3′) (RIBOBIO, Guangzhou, China) (final concentration: 100 nM) were respectively transfected into U251R and T98G cells using riboFECT^TM^ CP Transfection Agent (RIBOBIO) according to the manufacture’s instruction. At 24 h after siRNA transfection, cells were exposed to irradiation or other treatments.

### 4.5. Stable Transfection of shRNA

Cells were stably transfected with CD81 shRNA (Hanyin Biotechnology, Shanghai, China) (target sense: 5′-AATTGAAGACGAAGAGCAG-3′) or its negative control shRNA (target sense: 5′-TTCTCCGAACGTGTCACGT-3′). For this transfection, GBM cells were cultured with lentivirus at a multiplicity of infection (MOI) of 20, and polybrene (Hanyin Biotechnology) at a final concentration of 2 μg/mL was added into the culture medium to select the successfully transfected cells.

### 4.6. Subcellular Protein Extraction

U251R and T98G cells were plated on 10-cm dishes and incubated at 37 °C in 5% CO_2_. When cells reached about 70% confluence, a set of plates was exposed to IR. Cells were harvested at 0.5 h or 2 h post-IR. Control cells were not irradiated. Cell cytoplasmic protein and nuclear proteins were extracted using a subcellular protein fractionation kit (Beyotime Biotechnology) according to the manufacturer’s instruction, and their concentrations were determined with the bicinchoninic acid assay.

### 4.7. Western Blotting Assay

Protein samples were subjected to SDS-PAGE on 10% gel and transferred onto PVDF membranes, then probed with appropriate primary antibodies (CD81, 1:1000, Proteintech; γ-H2AX and Rad51, 1:1000, Cell Signaling Technology, Danvers, MA, USA) and then coated with second antibodies (1:5000, Signalway Antibody, College Park, ML, USA). The protein bands were detected using an ECL kit (BIO-RAD, Hercules, CA, USA) and their intensities were quantified using Quantity one software (BIO-RAD). Tubulin and Lamin A/C were used as loading control of whole cell protein and nuclear protein, respectively. The whole western blot figures can be found in the original images.

### 4.8. In Vivo Tumor Growth and Irradiation

A total of 40 five-week-old male BALB/c nude mice (18–20 g) were purchased from SIPPR/BK Lab. Animal Co. Ltd. (Shanghai, China) and maintained in a stable environment (23 °C, 12 h dark and 12 h light) for one week before experiments. For establishing the tumor xenograft model, 5 × 10^6^ U251R or T98G cells with or without CD81 interference were implanted subcutaneously into the lateral aspect of rear leg. When the tumor approached to 100 mm^3^ approximately, mice were randomized into IR group and non-IR control group (*n* = 5 for each group). Mice in IR group were given a total dose of 24 Gy X-rays irradiation in three consecutive days (8 Gy/day). The perpendicular diameter of each tumor was measured every three days with digital calipers, and the tumor volume was calculated using a formula (L × W^2^) × π/6, where L and W are the tumor’s length and width. Tumor growth delayed (TGD) was obtained for each treatment and was calculated as: TGD = [Ttv × 5] − [Tcv × 5], where Ttv × 5 and Tcv × 5 is the time to reach fivefold tumor volume increase compared to treatment start, based on an exponential growth fit in treated tumors (tv) and in untreated control tumors (cv), respectively. When Ttv × 5 was not reached, the volume of endpoint was used. Mice were sacrificed when tumors in non-IR group reached to 1400 mm^3^. Protein was extracted from dissected tumors using RIPA buffer and protease inhibitors. All animal experiments were approved by the Animal Ethics Committee of Fudan University (approval number 20171304A215).

### 4.9. Immunofluorescence Staining of BrdU, γ-H2AX and Rad51 Foci

Cells in 70–80% confluence were irradiated with 6 Gy X-rays. For BrdU assay, cells were incubated with 20 mM BrdU for one and half-cell cycles (36 h) prior to IR. Two hours after irradiation, cells were washed with cold PBS, fixed with 4% formaldehyde for 10 min, permeabilized with 0.5% Triton X-100 for 10 min and then incubated in 0.1% PBS-Tween solution containing 1% BSA, 10% normal goat serum, and 0.3M glycine for 1 h to block non-specific protein-protein interactions. Cells were then incubated with primary antibodies detecting γ-H2AX, Rad51, and BrdU (1:200, Cell Signaling Technology) overnight at 4 °C, and followed by Alexa Fluor 488 or 555-conjugated secondary antibody (1:1000, Cell Signaling Technology) for 1 h. DAPI was used to stain the cell nuclei at a concentration of 1.43 μM. Cell fluorescence image was photographed with a high content screening system (ImageXpress Micro 4, Molecular Devices, San Jose, CA, USA).

### 4.10. Dual Immunofluorescence Assay of CD81 and Rad51

In order to observe nuclear membrane proteins clearly, cells in 24-well plate were treated with hypotonic solution of 0.075 M potassium chloride (KCl) at 37 °C for 25 min, and cytoplasm was washed out with PBS. Then the cells were fixed in 4% formaldehyde followed by permeabilization treatment with 0.5% Triton X-100 for 10 min, blocked with 0.1% PBS-Tween solution for 1 h, and probed with rabbit-anti-Rad51 antibody for 12 h and mouse-anti-CD81 antibody (1:200, Proteintech, Rosemont, IL, USA) for another 12 h, followed by incubation with Alexa Fluor 555 goat anti-rabbit antibody for 1 h and Alexa Fluor 488 goat anti-mouse antibody for another 1 h. After washing with pre-cold PBS triply, cell nuclei were stained with DAPI at a concentration of 1.43 μM for 10 min.

### 4.11. Flow Cytometry Assay of Cell Cycle

The cell cycle distribution was analyzed with flow cytometry assay. Briefly, cells in exponential growth were seeded into 6-well plate (1 × 10^6^ cells per well) and irradiated with 6 Gy X-rays. At 24 h after IR, cells were collected, fixed with 70% ethanol overnight at −20 °C, stained with propidium iodide (PI) for 30 min at 4 °C in darkness, then subjected to a flow cytometer (CytoFLEX S, Beckman Coulter, Brea, CA, USA). The proportion of cells in G0/G1, S, and G2/M phase was calculated using Flowjo software (BD Biosciences).

### 4.12. Rad51 Inhibitor Treatment

B02 (MedChemExpress, Monmouth Junction, NJ, USA) was freshly stocked in dimethyl sulfoxide (DMSO) at 10 mM. U251R and T98G cells with or without CD81 siRNA interference were treated with 10 μM B02 or it control (1‰ DMSO) for 24 h and then followed by 6 Gy irradiation. Control groups were not irradiated. 

### 4.13. Statistical Analysis

All results were presented as the mean ± SE of at least three independent experiments. One-way ANOVA test for the statistical difference of cell survival, tumor growth delay, and clinical data, or unpaired t-test for Western blot and immunofluorescence staining results was used with GraphPad Prism 8.0 (GraphPad Software Inc., La Jolla, CA, USA). All statistical tests were two tailed, and *p* < 0.05 was considered significantly different between indicated groups.

## 5. Conclusions

Our study not only discovered novel roles of nuclear membrane CD81 in mediating nuclear translocation of Rad51, promoting HRR process in IR-induced DNA repair, and regulating radioresistance of GBM cells, but also lunched a clinical opportunity of treating GBM with a combination strategy of CD81 inhibitor and radiotherapy.

## Data Availability

The data presented in this study are available on request from the corresponding author upon reasonable request.

## Supplementary Materials

The following are available online at https://www.mdpi.com/article/10.3390/cancers13091998/s1, Figure S1. Efficiency of siCD81 and shCD81 transfection in GBM cells. Figure S2. Suppression of CD81 reduced G2/M arrest in GBM cells exposed to IR.
