# Supplementary material for: CD81 Enhances Radioresistance of Glioblastoma by Promoting Nuclear Translocation of Rad51
Source: Cancers (Basel). 2021 Apr 21;13(9):1998. doi: 10.3390/cancers13091998 (PMC8122253; doi:10.3390/cancers13091998)

Western blots in the article and original blots

Figure.2A

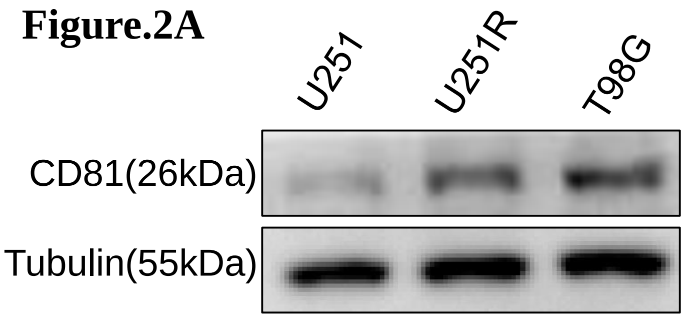

Original uncropped western

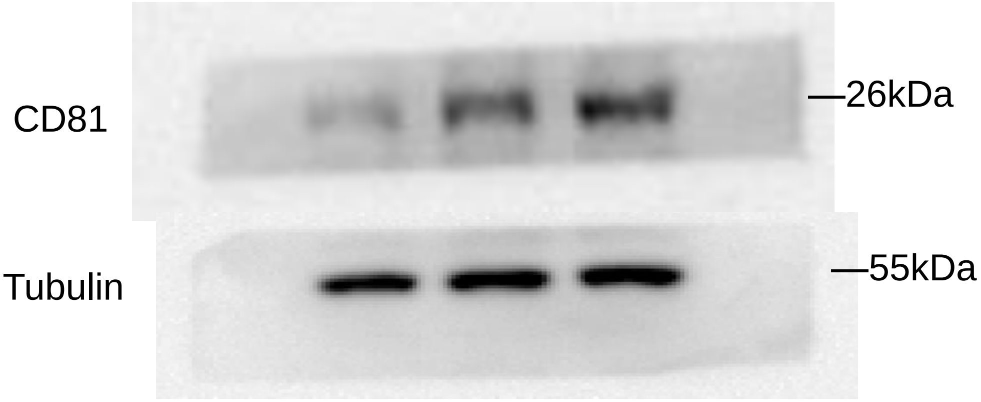

Figure.3A

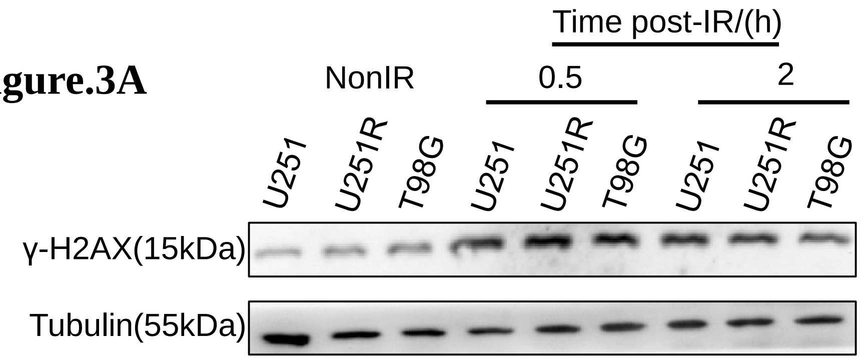

Original uncropped western

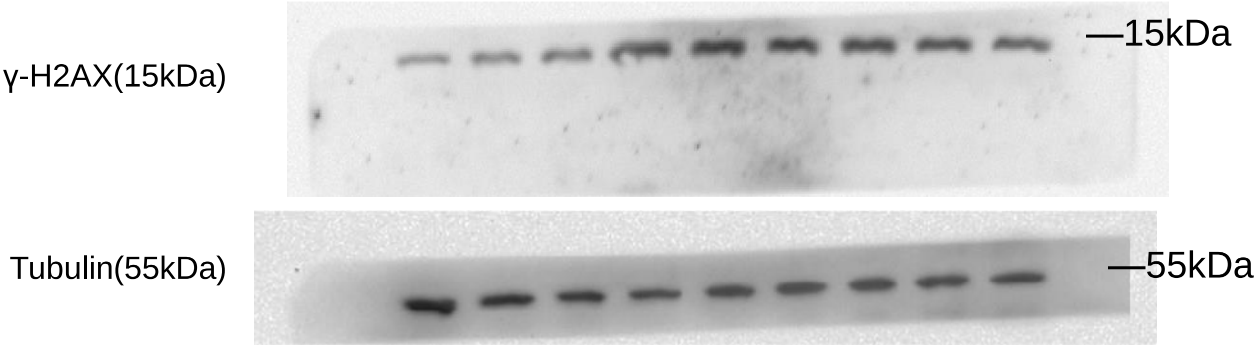

Figure.3B

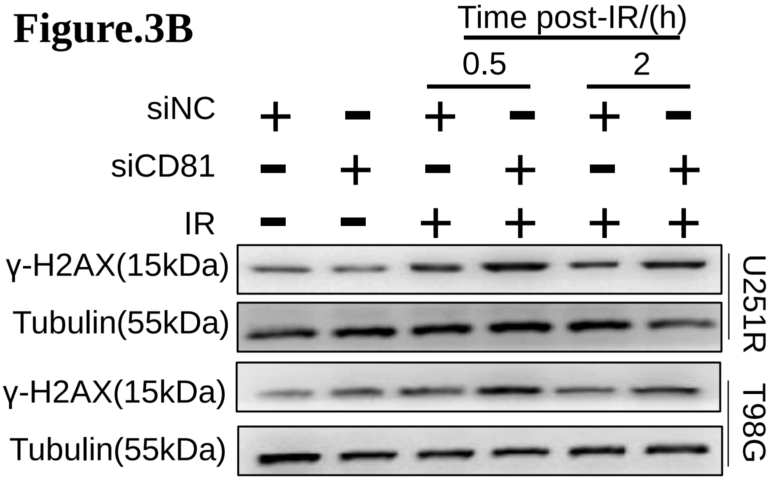

Original uncropped western

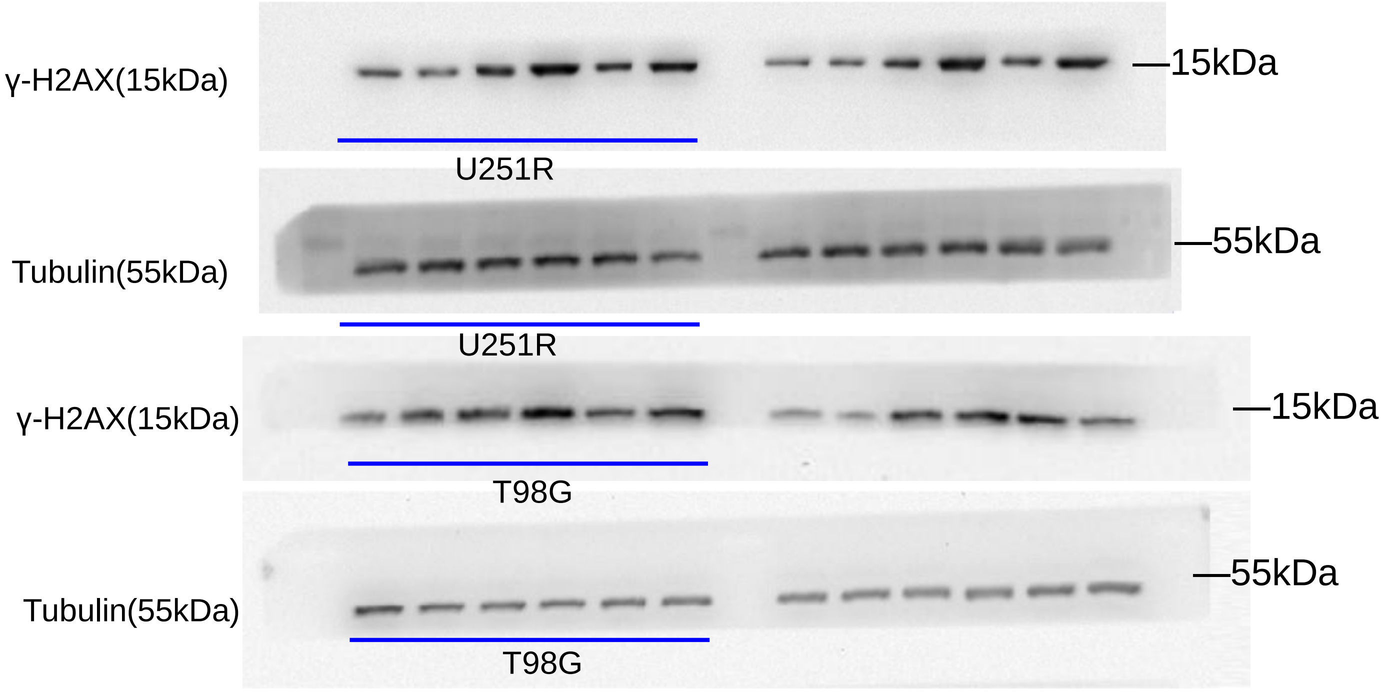

Figure.4A

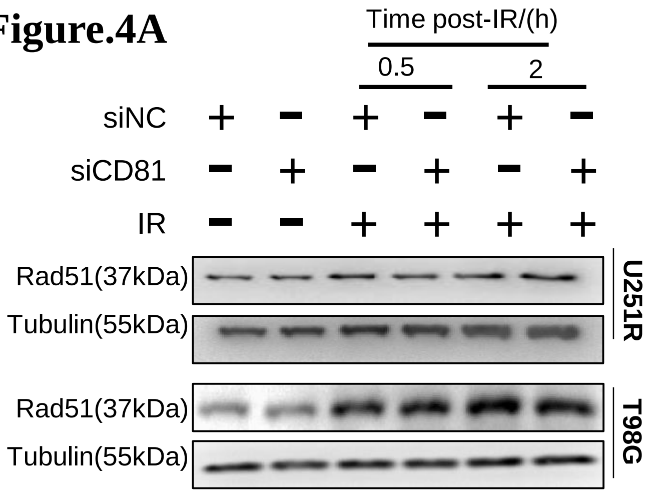

Original uncropped western

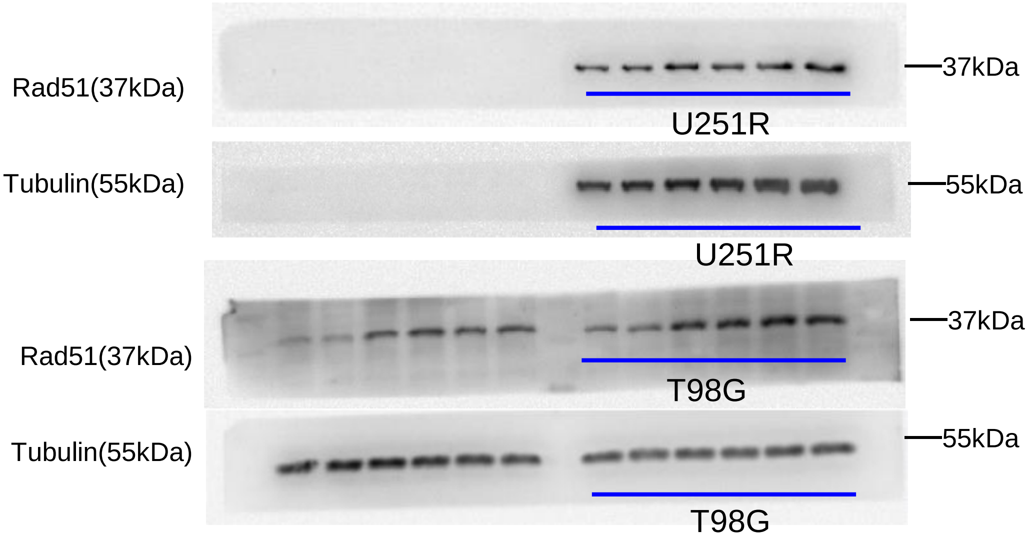

Figure.4B

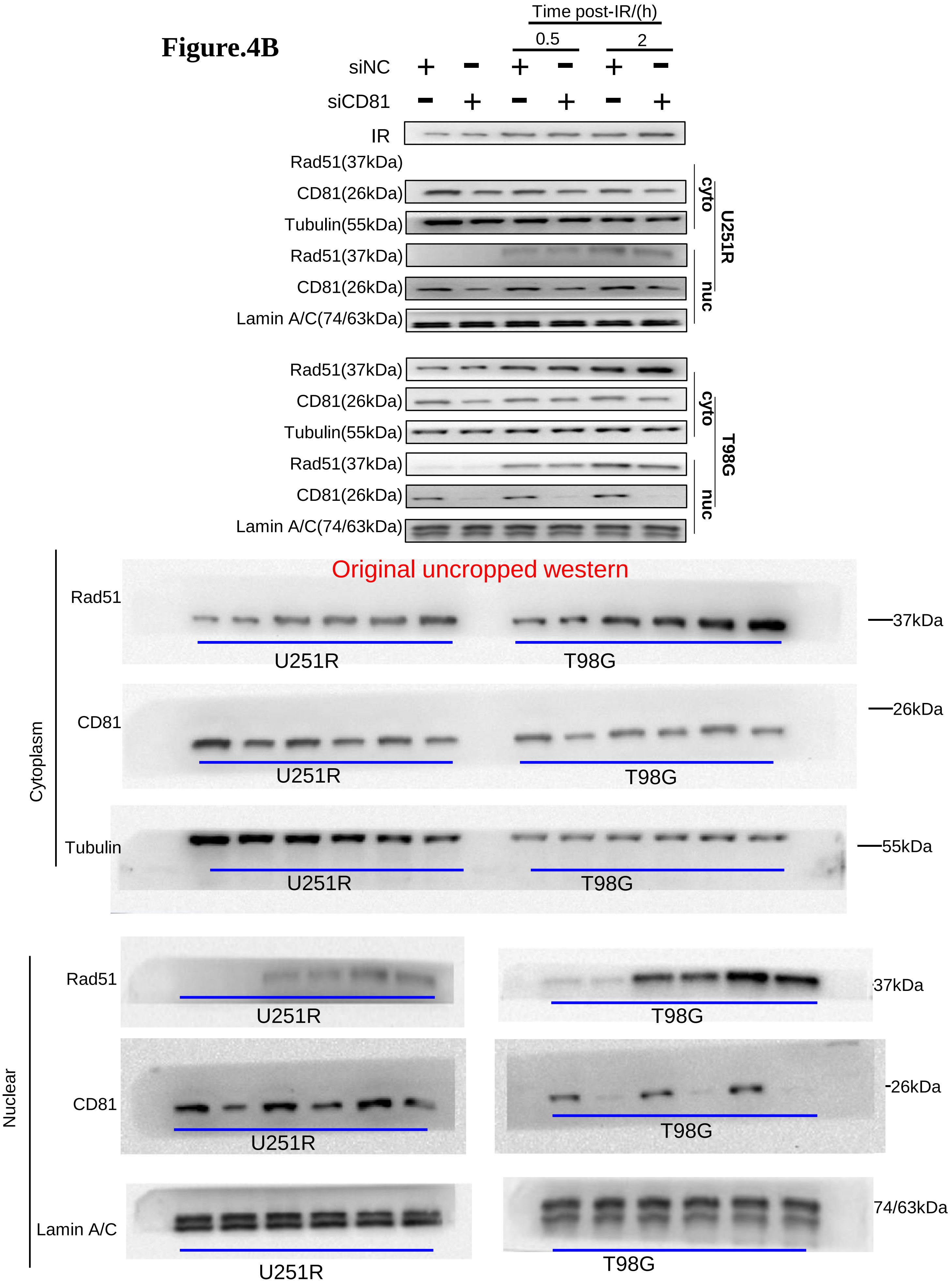

Time post-IR/(h)
0.5
2
siNC
siCD81
IR
Rad51(37kDa)
CD81(26kDa)
Tubulin(55kDa)
Rad51(37kDa)
CD81(26kDa)
Lamin A/C(74/63kDa)
cyto
nuc
U251R
Rad51(37kDa)
CD81(26kDa)
Tubulin(55kDa)
Rad51(37kDa)
CD81(26kDa)
Lamin A/C(74/63kDa)
cyto
nuc
T98G
Original uncropped western
Cytoplasm
Rad51
37kDa
U251R
T98G
CD81
26kDa
U251R
T98G
Tubulin
55kDa
U251R
T98G
Nuclear
Rad51
U251R
T98G
37kDa
CD81
U251R
T98G
26kDa
Lamin A/C
U251R
T98G
74/63kDa

Figure.5B

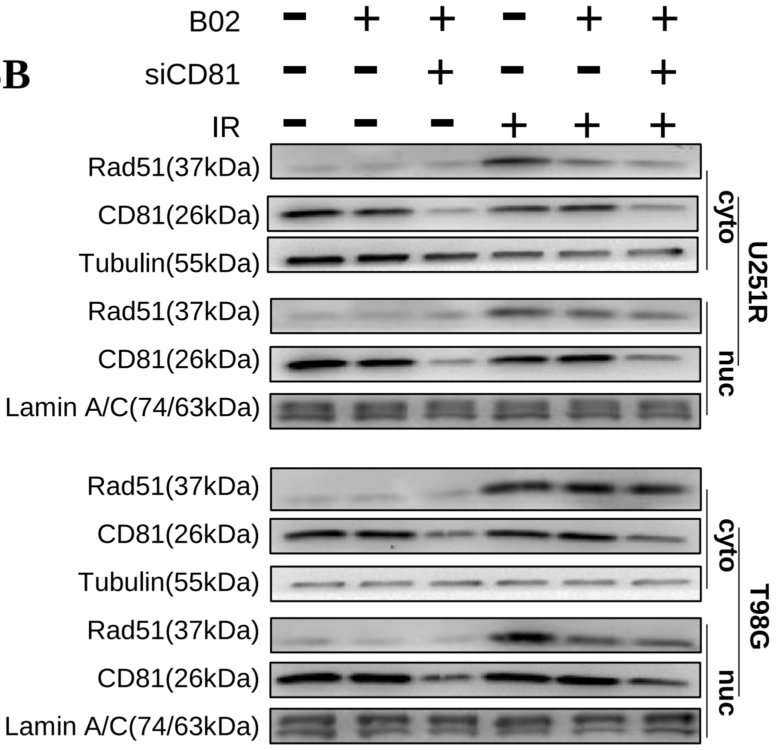

Original uncropped western

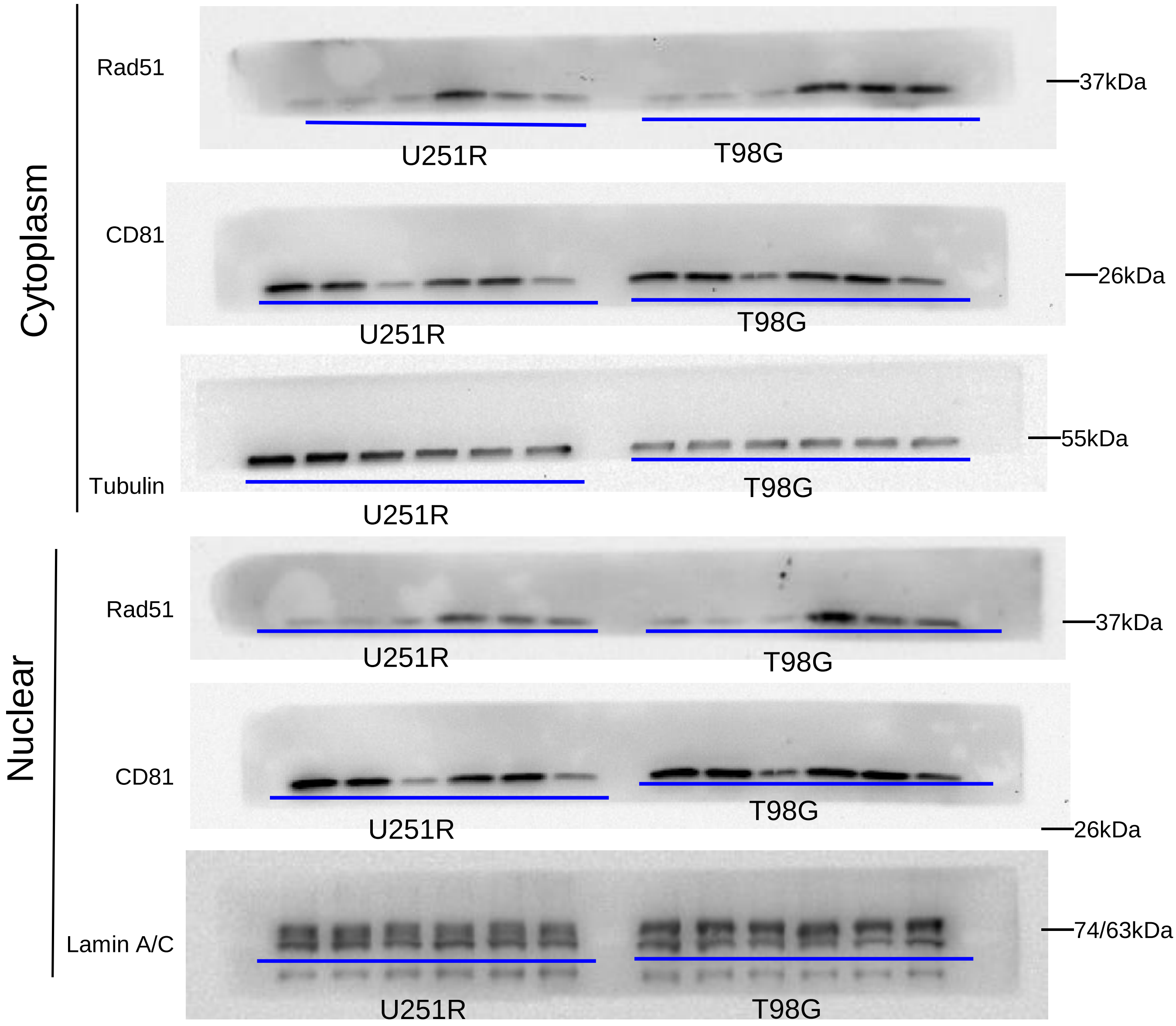

Figure.6B

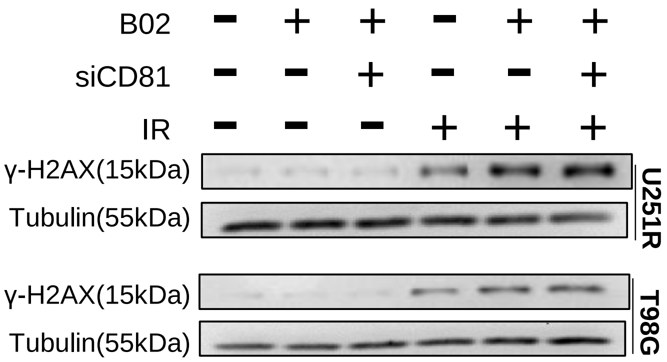

Original uncropped western

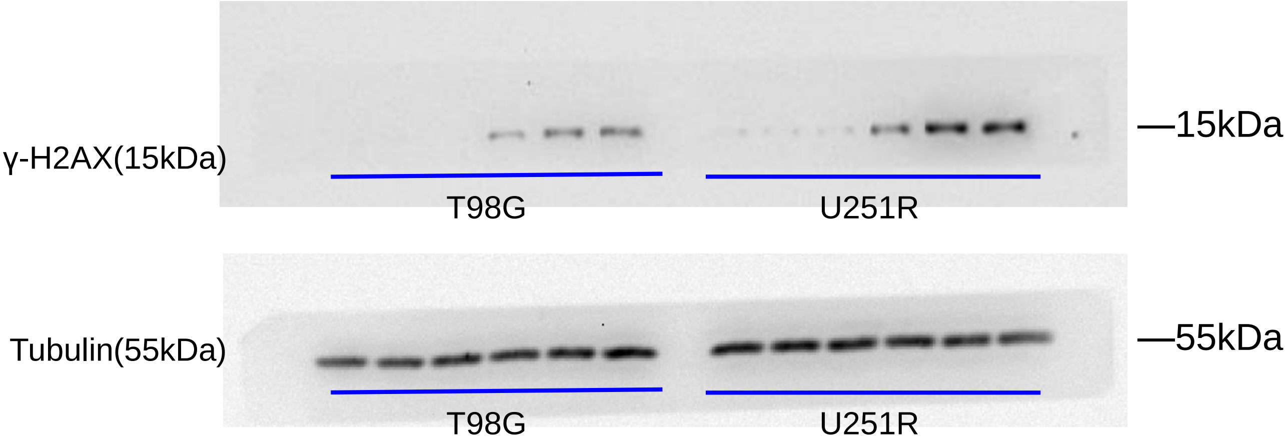

Supplement Figure S1

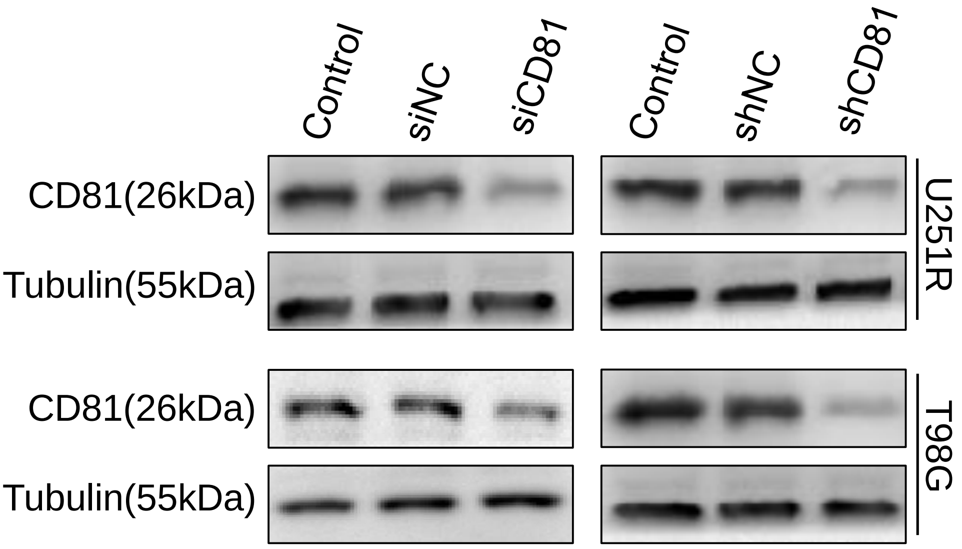

Original uncropped western

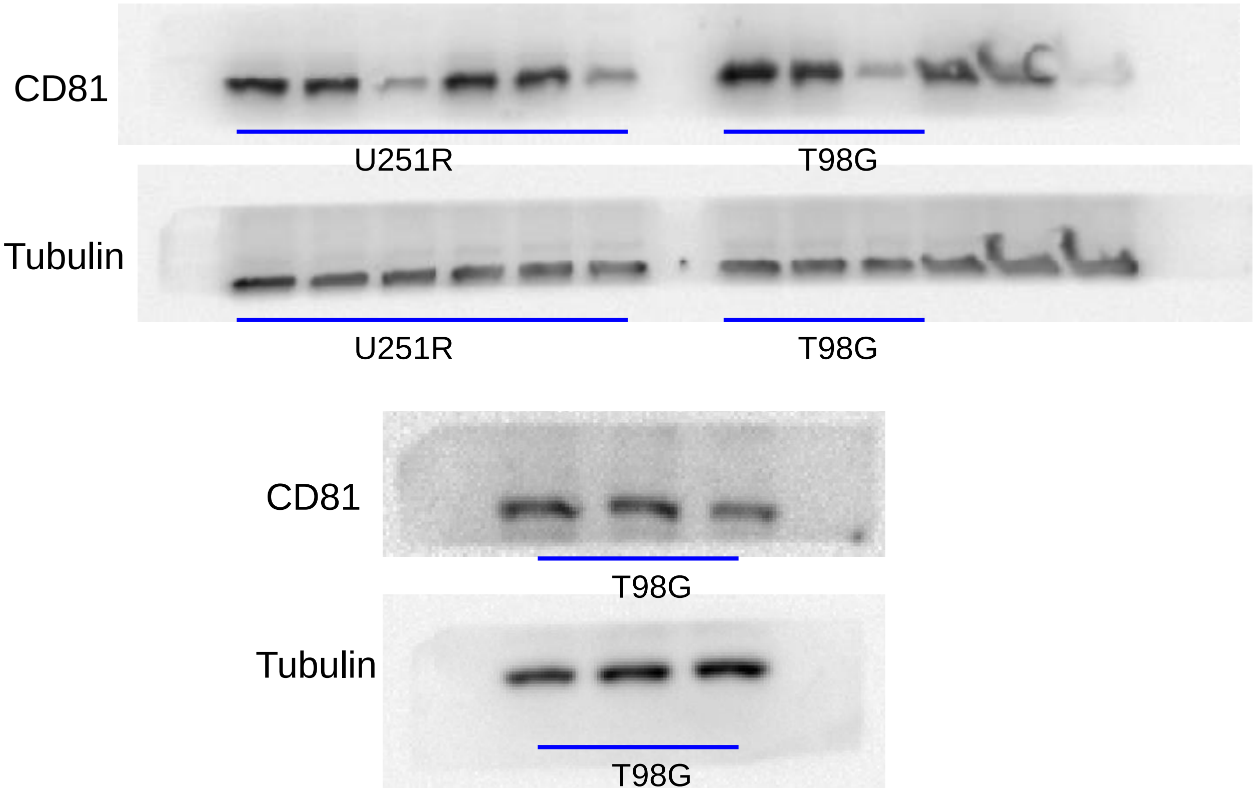

Supplement: Supplementary file 1 [file cancers-13-01998-s001.zip › supplementary/cancers-1103327-original-images.pdf]
