# Supplementary material for: CD81 Enhances Radioresistance of Glioblastoma by Promoting Nuclear Translocation of Rad51
Source: Cancers (Basel). 2021 Apr 21;13(9):1998. doi: 10.3390/cancers13091998 (PMC8122253; doi:10.3390/cancers13091998)
Supplement: Supplementary file 1 [file cancers-13-01998-s001.zip › supplementary/cancers-1170139-supplementary-done.pdf]

cancers MDPI

Supplementary material

CD81 Enhances Radioresistance of Glioblastoma by Promoting Nuclear Translocation of Rad51

Wang Zheng, Qianping Chen, Hongxia Liu, Songling Hu, Yuchuan Zhou, Yang Bai, Jianghong Zhang, Yan Pan and Chunlin Shao

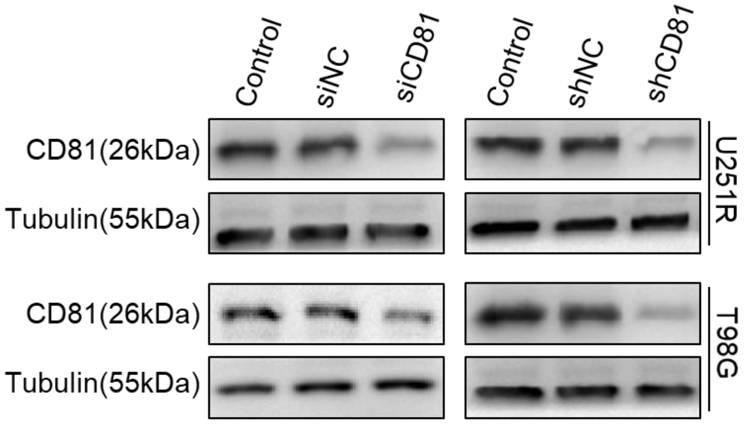

Figure S1. Efficiency of siCD81 and shCD81 transfection in GBM cells. Expression of CD81 protein in U251R and T98G cells transfected with siNC, siCD81, shNC and shCD81 were detected by western blot, respectively. Tubulin was used as loading control.

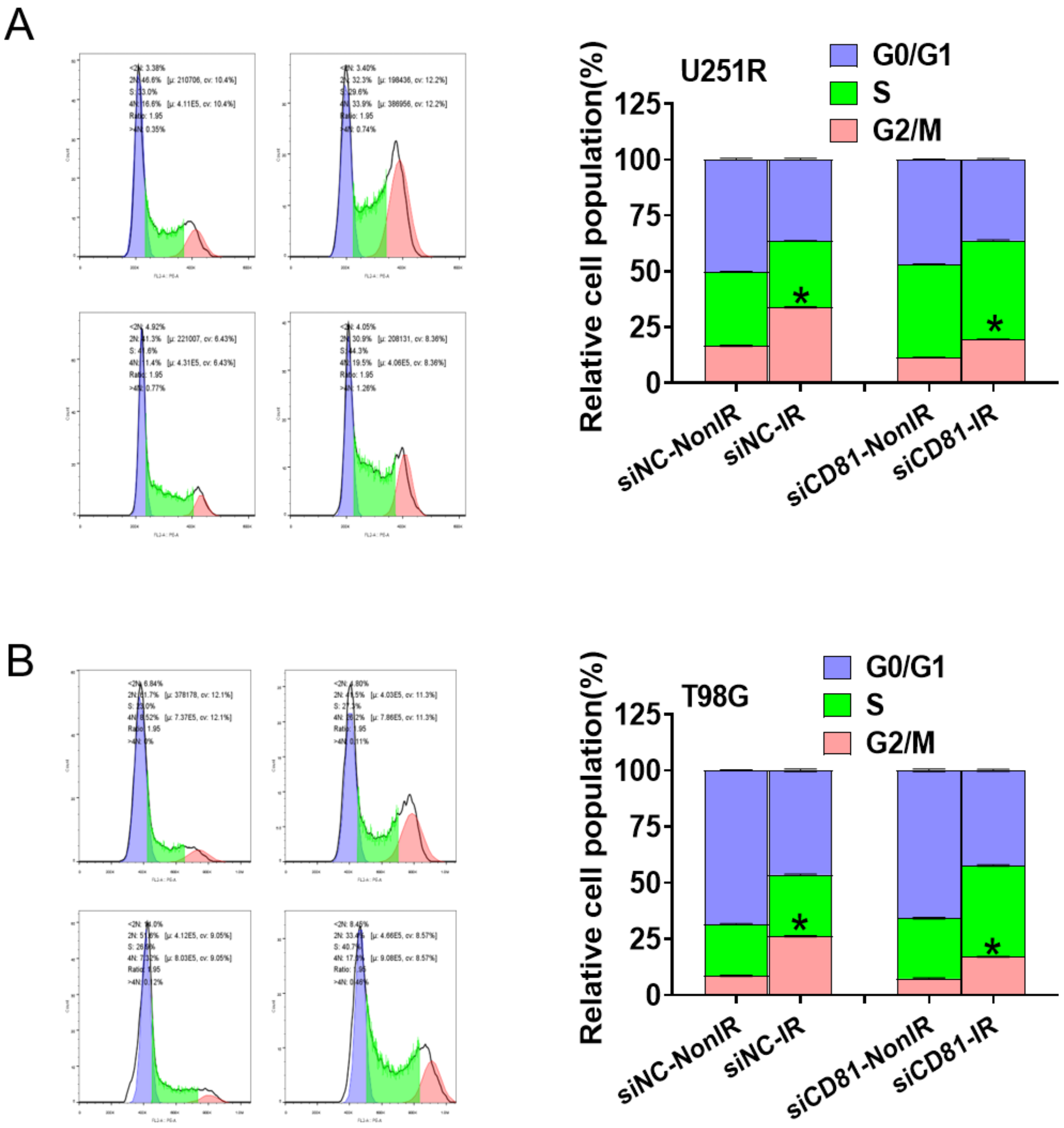

Figure S2. Suppression of CD81 reduced G2/M arrest in GBM cells exposed to IR. Cell cycle distributions of U251R (**A**) and T98G (B) cells transfected with siNC or siCD81 were measured by flow cytometry at 24 h after 6 Gy X-rays. Blue, G0/G1 phase. Green, S phase. Pink, G2/M phase. *, $P < 0.05$ in compassion with G2/M phase of non-IR cells.
